# A comprehensive evaluation of the associations between 12 composite inflammatory indices and all-cause mortality after stroke: a multicohort study

**DOI:** 10.3389/fnagi.2025.1754095

**Published:** 2026-01-09

**Authors:** Longyi Zheng, Rundong He, Shuang Tang, Jia Duan, Wenli Xing, Ao Qian

**Affiliations:** 1 Department of Radiology, Suining Central Hospital, Suining, Sichuan, China; 2 Department of Cerebrovascular Disease, Suining Central Hospital, Suining, Sichuan, China

**Keywords:** composite inflammatory index, cross-sectional, mortality, national health and nutrition examinationsurvey, neutrophil-to-lymphocyte ratio, retrospective, stroke

## Abstract

**Background:**

Inflammation plays a critical role in post-stroke mortality. However, identification of robust and generalizable inflammatory biomarkers for post-stroke mortality remains a challenge. We conducted a comprehensive analysis of various composite inflammatory indices, evaluating the associations between these indices and post-stroke mortality by examining two cohorts, to discover trans-situationally robust indices.

**Methods:**

Data were sourced from the National Health and Nutrition Examination Survey (NHANES) circles of 1999–2010 and 2015–2018, as well as from our stroke center. Twelve composite inflammatory indices were calculated based on peripheral blood cell, C-reactive protein, and albumin. The correlations between these indices and post-stroke mortality were evaluated using multivariate Cox proportional hazards regression analyses, with the false discovery rate (FDR) correction applied for multiple testing. The neutrophil-to-lymphocyte ratio (NLR), which demonstrated consistent significance in both NHANES and clinical cohorts, was further subjected to subgroup analyses to elucidate its relationship with post-stroke mortality across various conditions.

**Results:**

This study included 1,152 participants from NHANES cohort, followed until December 31, 2019, and 2,540 patients with acute ischemic stroke (AIS) from the clinical cohort with 90-day follow-up. The NLR, whether treated as a continuous or categorical variable (classified into tertiles), was significantly associated with mortality in both NHANES (per unit increase: hazard ratio [HR] 1.101, 95% confidence interval [CI] 1.043–1.163, *P_-FDR_* = 0.001; T3 vs. T1: HR 2.002, 95% CI 1.555–2.577, *P_-FDR_* < 0.001) and clinical cohort (per unit increase: HR 1.023, 95% CI 1.010–1.037, *P_-FDR_* = 0.002; T3 vs. T1: HR 1.939, 95% CI 1.342–2.804, *P_-FDR_* = 0.009). Subgroup analyses revealed a significant interaction between NLR and time from AIS onset to admission in clinical cohort (*P* for interaction = 0.017), demonstrating the association was particularly strong in patients admitted within 24 h of AIS onset (HR 1.024, 95% CI 1.011–1.038, *p* < 0.001).

**Conclusion:**

The NLR may serve as a generalizable biomarker of post-stroke mortality assessment across both community and clinical settings. The correlation with mortality is pronounced in patients during early stage of AIS, underscoring the time-sensitive prognostic value of NLR.

## 1 Introduction

As a major global health issue, stroke is characterized by high disability and mortality, imposing substantial burden on society (Qian et al., 2025). Although notable advancements have been achieved in stroke therapy, high rate of adverse outcome continues to pose a formidable challenge (Duan et al., 2025). Thus, identification the factors associated with post-stroke mortality is a vital step for developing strategies that improve survival and overall prognosis.

Inflammation plays a critical role in the pathophysiology of stroke, involving in its onset, progression, and resolution, as well as in the remodeling and repair of ischemic tissue (Qian et al., 2024). However, a persistent or dysregulated inflammatory response may evolve into a chronic state that exacerbates long-term tissue damage (Huang et al., 2024). Composite inflammatory indices, calculated from common peripheral blood parameters, provides a convenient and accessible measure of systemic inflammatory status (Zhou et al., 2025). Recent studies have confirmed associations between these indices and long-term mortality after stroke in community-based population (Xu et al., 2025; Yang and Wang, 2025). Furthermore, clinical studies have also demonstrated their correlation with short-term mortality in patients with acute ischemic stroke (AIS) (Wu and Chen, 2023; Qian et al., 2024). However, the variety of composite inflammatory indices proposed by previous reports creates substantial confusion for clinicians and researchers in selecting an appropriate index, which is highly contingent on specific circumstances, for prognostic evaluation, thereby increasing the burden on stroke management and hampering the generalizability of these indices. Critically, this confusion is compounded by a more fundamental gap that the lack of systematic evidence regarding the robustness of these indices across different stages of stroke. Evidence derived from either clinical cohort or community-based population remains unclear whether a biomarker that shows association with mortality in the acute phase retains its prognostic value in the chronic phase, and vice versa. This lack of trans-situational validation may limit the identification of a universally applicable inflammatory biomarker. To address this gap, we conducted this study to investigate the associations between 12 common composite inflammatory indices, including systemic immune-inflammation index (SII), neutrophil-to-lymphocyte ratio (NLR), platelet-to-lymphocyte ratio (PLR), lymphocyte-to-monocyte ratio (LMR), neutrophil-to-platelet ratio (NPR), systemic immune-inflammation response index (SIRI), platelet-to-albumin ratio (PAR), neutrophil percentage-to-albumin ratio (NPAR), C-reactive protein to lymphocyte ratio (CLR), C-reactive protein-albumin-lymphocyte index (CALLY), pan-immune-inflammation value (PIV), and hemoglobin-albumin-lymphocyte-platelet score (HALP), and all-cause mortality after stroke across two cohorts, with the aim of identifying potential trans-situational inflammatory biomarkers.

## 2 Methods

### 2.1 Study design and population

This study was conducted in two phases. In the initial phase, we analyzed data from the National Health and Nutrition Examination Survey (NHANES), a biennial, cross-sectional program that assesses the health and nutritional status of the noninstitutionalized civilian population in the United States. Its design employs a complex, stratified, multistage probability sampling strategy. Data collection, which has been extensively documented, encompasses interviews, physical examinations in mobile examination centers (MECs), and laboratory tests (Mao et al., 2024; Cheng et al., 2023). The survey is administered by the National Center for Health Statistics (NCHS) under the Centers for Disease Control and Prevention (CDC) with approval from the NHANES Institutional Review Board.^1^ All participants provided written informed consent. Our analysis utilized only the de-identified and publicly available data from NHANES in strict compliance with its data use regulations.^2^ Due to the absence of C-reactive protein (CRP) data in the 2011–2014 circles, our analysis was restricted to data from the 1999–2010 and 2015–2018 survey cycles. Exclusion criteria were as follows: (1) with age < 20 years; (2) who were pregnant or had cancer; (3) without diagnosis of stroke; (4) without data of peripheral blood cell counts, CRP, or albumin; and (5) lack of mortality data.

In the subsequent phase, the data from our stroke center were reviewed to establish a clinical cohort after approving by our institutional ethics committee (approve number: KYLLKS20250217). Consecutive patients diagnosed with AIS at Suining Central Hospital from January 2021 to December 2024 were systematically screened for eligibility. The inclusion criteria included: (1) diagnosis of AIS confirmed by magnetic resonance imaging (MRI) or computed tomography (CT); (2) age > 18 years; (3) time from symptom onset (or the last known well) to admission within 72 h. Furthermore, some patients who achieved obvious improvement of neurological symptoms after intravenous thrombolysis were also diagnosed with AIS, despite the absence of definitive cerebral infarction lesion on subsequent neuroimaging. The exclusion criteria were as follows: (1) missing data on components of composite inflammatory indices or loss to follow-up within 90 days after AIS onset; (2) presence of decompensated or progressive comorbidities at AIS onset; (3) having malignancy, active infection, or immunologic disease at admission; (4) concomitant conditions requiring corticosteroid or immunosuppressant therapy; (5) history of prior stroke with obvious residual functional impairment. In addition, patients who underwent mechanical thrombectomy (MT) for acute large occlusion or decompressive craniectomy (DC) for massive cerebral edema were also excluded, as these major interventions represent substantial confounding factors for assessing the prognostic value of baseline inflammatory biomarkers.

### 2.2 Definition of the composite inflammatory indices and mortality

The composite inflammatory indices analyzed in this study were selected based on previous studies (Zhou et al., 2025; Chen et al., 2024a; Lv et al., 2023), and were derived from peripheral blood parameters, including neutrophil, lymphocyte, monocyte, platelet, hemoglobin, CRP, and albumin. The formulae of calculation were listed as follows: SII = neutrophil count * platelet count / lymphocyte count; NLR = neutrophil count / lymphocyte count; PLR = platelet count / lymphocyte count; LMR = lymphocyte count / monocyte count; NPR = neutrophil count / platelet count; SIRI = neutrophil count * monocyte count / lymphocyte count; PAR = platelet count / albumin level; NPAR = neutrophil percentage / albumin level; CLR = CRP level / lymphocyte count; CALLY = albumin level * lymphocyte count / (CRP level *10); PIV = neutrophil count * monocyte count * platelet count / lymphocyte count; HALP = albumin level * hemoglobin level * lymphocyte count / platelet count.

In the NHANES cohort, data on all-cause mortality were linked from the death linkage files until December 31, 2019. The follow-up time was calculated from the baseline examination date to the date of death or the end of the follow-up period. In the clinical cohort, the mortality status at 90 days after AIS onset was assessed by qualified personnel or physicians through in-person interviews at the clinic or telephone.

### 2.3 Data collection

#### 2.3.1 NHANES database cohort

Stroke was identified through a questionnaire by participants who answered “yes” to the question, “Have you ever been told by a physician or a health professional that you had stroke.” Despite the absence of stroke subtype information in the NHANES database, it is likely that the majority of participants in our study had ischemic stroke, given that ischemic stroke accounts for 80–85% of all stroke cases and is closely linked to chronic inflammation (Ananth et al., 2023; Mao et al., 2024).

Age, sex, race/ethnicity, educational level, poverty income ratio, smoking status, alcohol consumption, and comorbidities were ascertained via standardized questionnaires and interviews. All physical examinations and laboratory tests were administered by qualified personnel in the MEC. Race/ethnicity was categorized into five groups: Mexican American, Other Hispanic, Non-Hispanic White, Non-Hispanic Black, and Other Races. Educational level was classified as below high school, high school, or above high school. Smoking was defined as having smoked more than 100 cigarettes in one’s lifetime, while alcohol drinkers were identified as those who consumed at least 12 drinks per year (Chen et al., 2016; Liu et al., 2023). Body mass index (BMI) was calculated as weight in kilograms divided by height in meters squared (kg/m^2^). Systolic and diastolic blood pressure (SBP/DBP) were measured by experienced clinicians following a standardized protocol using a mercury sphygmomanometer; three consecutive readings were taken at 30-s intervals, and the mean value was designated as the blood pressure of participants (Mao et al., 2024). Hypertension, hyperlipidemia and diabetes mellitus were defined as self-reported diagnosis or current use of relevant drugs. Moreover, for diabetes mellitus, an alternative definition of glycated hemoglobin (HbA1c) level more than 6.5% was also applied (Mao et al., 2024). Laboratory parameters comprised complete blood count, CRP, albumin, alanine aminotransferase (ALT), aspartate aminotransferase (AST), HbA1c, triglycerides (TG), total cholesterol (TC), low-density lipoprotein cholesterol (LDL-C), and high-density lipoprotein cholesterol (HDL-C). To calculate the estimated glomerular filtration rate (eGFR), the investigators applied the Chronic Kidney Disease Epidemiology Collaboration (CKD-EPI) formula, which involves age, sex, race/ethnicity, and serum creatinine to enhance the accuracy across diverse population (Levey et al., 2009).

#### 2.3.2 Clinical cohort

Patient management was conducted in accordance with the Chinese Stroke Association guidelines for cerebrovascular disease (Liu et al., 2020). The following data were reviewed: demographics, time from onset to admission, use of intravenous thrombolysis, medical history, neurological deficit severity assessed by the National Institutes of Health Stroke Scale (NIHSS), stroke etiology classified via the Trial of ORG 10172 in Acute Stroke Treatment (TOAST) criteria (Adams et al., 1993), laboratory parameters, and follow-up information.

Smokers were defined as individuals who had smoked at least one cigarette daily for at least 1 year (Sun et al., 2017). Alcohol drinker was characterized by a daily intake of at least 80 grams of alcohol (Chen et al., 2023). Blood samples were obtained from all AIS patients immediately upon admission for laboratory tests of complete blood count, CRP, glucose, coagulation profile, hepatic and renal function, and electrolytes. Lipid profile and HbA1c were measured using fasting blood samples collected on the next morning following admission.

### 2.4 Statistical analysis

The selection of variables was based on previous literature and our experience. Variables with missing data exceeding 20% were excluded from the analysis, whereas others were imputed through multiple imputation for missing values. Survey weights were employed in the analyses of NHANES cohort for its stratified multistage design. All the continuous variables were expressed as median (interquartile range [IQR]) due to the skewed distribution confirmed by the Shapiro-Francia test. Group comparisons for continuous variables were performed using the weighted Mann–Whitney U test for the NHANES cohort, and the Mann–Whitney U test for the clinical cohort. Categorical variables are summarized as percentages (95% confidence interval [CI]) and compared using the weighted chi-square test in the NHANES cohort, while in the clinical cohort, they are presented as frequency (percentage) and compared using the standard chi-square test. The discrimination ability was evaluated by receiver operating characteristic (ROC) curves, and quantified with area under the curve (AUC) calculations. We analyzed the composite inflammatory indices as continuous and categorical variables (grouped into tertiles, T1-T3, with T1 as the reference) in both cohorts. Cox proportional hazards regression analyses were then employed to quantify the hazard ratios (HRs) and 95% CI for their associations with all-cause mortality. Multivariate regression models applied to the NHANES cohort included Model 1: no adjustment; Model 2: adjusted for age, sex, and race; Model 3: adjusted for age, sex, race, education level, smoking, drinking, BMI, hypertension, diabetes mellitus, hyperlipidemia, eGFR, AST, TG, TC, and LDL-C. For the clinical cohort, several models were also included (Model 1: no adjustment; Model 2: adjusted for age, sex, and time from onset to admission; Model 3: adjusted for age, sex, time from onset to admission, history of diabetes mellitus, coronary heart disease, atrial fibrillation and congestive heart failure, previous stroke, anticoagulant therapy at admission, TOAST classification, initial NIHSS score, sodium, chlorine, prothrombin time (PT), fibrinogen, international normalized ratio (INR), AST, creatinine, cystatin C, and LDL-C). A linear trend test was performed by entering the median value of each tertile as a continuous variable. To control for the increased risk of false positives arising from multiple comparisons, we applied the Benjamini-Hochberg method for false discovery rate (FDR) correction to the association models. The potential nonlinear relationships between the composite inflammatory indices and mortality were examined using restricted cubic splines (RCS), with adjustment for the covariates in Model 3. The Kaplan–Meier curves were plotted to visualize survival differences across the tertile of the indices.

The NLR, which demonstrated a significant association with post-stroke mortality in both NHANES and clinical cohorts, was selected for further subgroup analyses. Planned stratification was performed by sex (female or male), age (> 65 or ≤ 65 years), BMI (≥ 30 or < 30), hypertension (yes or no), diabetes mellitus (yes or no), hyperlipidemia (yes or no), and race (white, black, or other) in the NHANES cohort, and by sex (female or male), age (≥ 70 or < 70 years), time from onset to admission (≤ 24 or 24–72 h), history of hypertension (yes or no) and diabetes mellitus (yes or no), initial NIHSS score (≤ 5 or > 5), and TOAST classification (small-artery occlusion, large-artery atherosclerosis, or cardioembolism) in the clinical cohort. Likelihood ratio tests were used to assess the interaction effects between NLR and these stratification variables. The HRs with 95% CI within each subgroup were calculated using the same covariate adjustments as in Model 3. In addition, to assess the predictive performance of the NLR in clinical utility, we appended it to Model 2, Model 3, and NIHSS score to calculate the difference in model performance using C-statistics, the category-free net-reclassification index (NRI), and integrated discrimination improvement (IDI) in clinical cohort. All statistical analyses were performed using R software (version 4.4.2, https://www.R-project.org/). A two-tailed *p* value < 0.05, and *P_-FDR_* value < 0.05 for multivariable analyses were considered statistically significant.

## 3 Results

### 3.1 NHANES cohort

**Figure 1:**
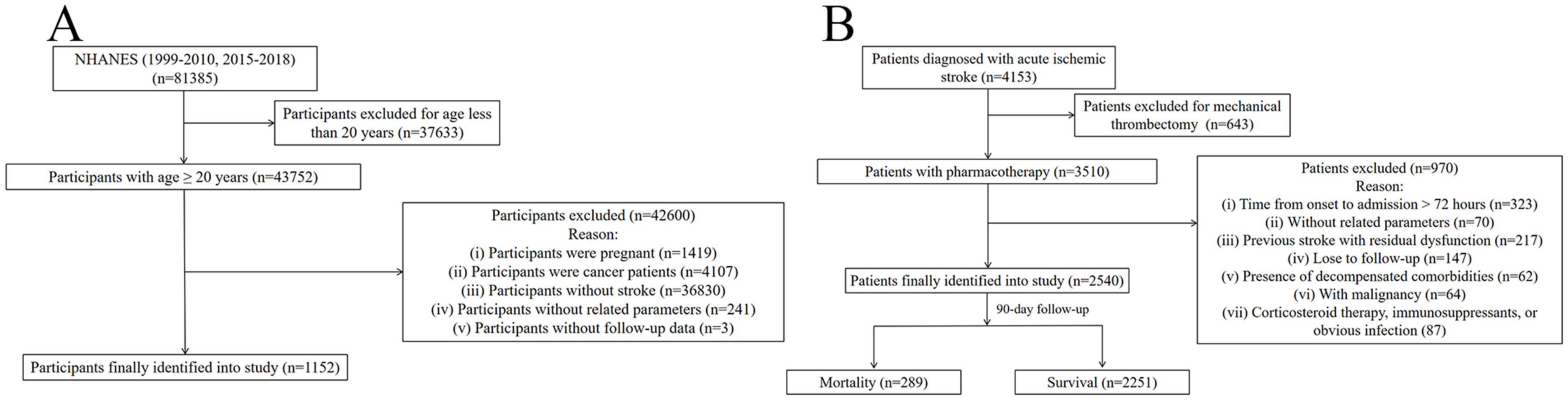
Process of patient selection. **(A)** The NHANES cohort. **(B)** The clinical cohort.

**Figure 2:**
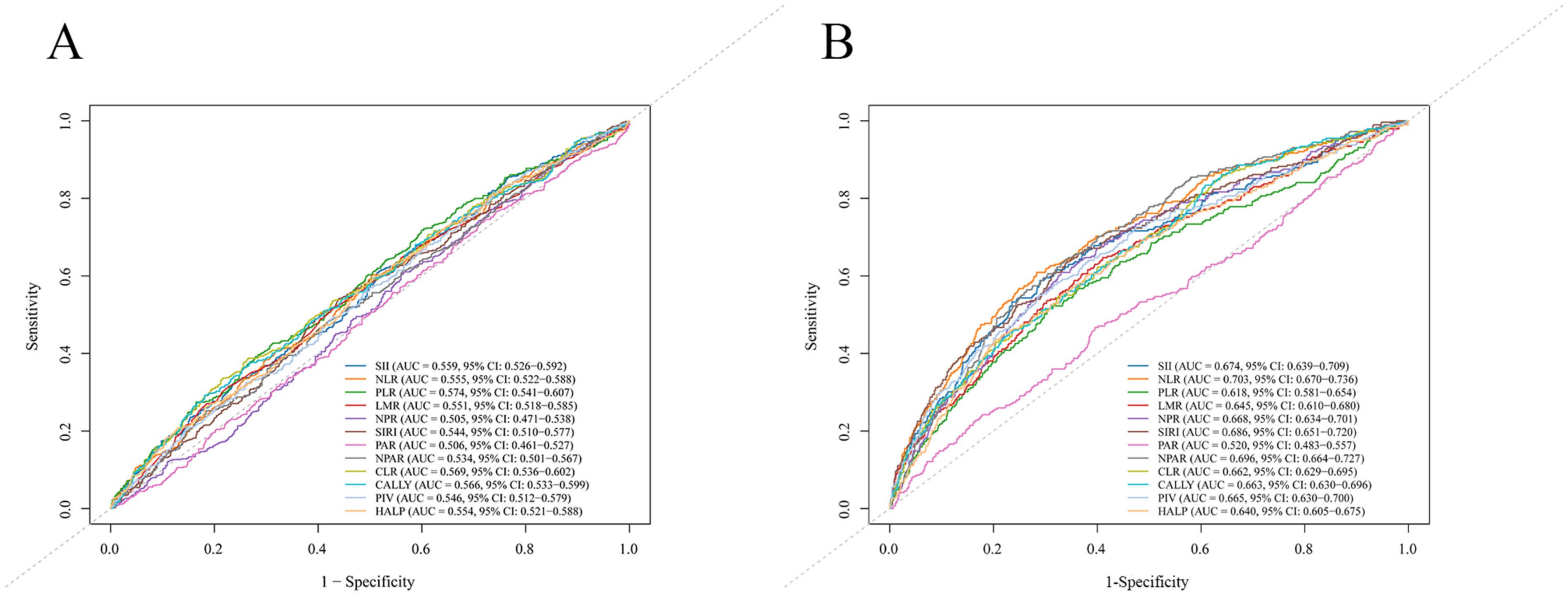
Receiver operating characteristic curves demonstrated the discrimination ability of composite inflammatory indices for post-stroke mortality in NHANES **(A)** and clinical cohorts **(B)**.

After the exclusion of ineligible participants, 1,152 individuals were included in the final analysis (Figure 1A), with their baseline characteristics summarized in Table 1. Compared with survivors, non-survival participants were older, had lower educational levels, and higher SBP. Regarding laboratory parameters, these participants exhibited lower lymphocyte count and eGFR, alongside elevated levels of CRP, TG, TC, and LDL-C (all *p* < 0.05). Among the composite inflammatory indices, all except for NPR, PAR, and NPAR showed significant differences between mortality and survival groups (all *p* < 0.05) (Table 2). However, ROC curve revealed that all composite inflammatory indices demonstrated limited discrimination ability for mortality, with all AUC values below 0.6 (Figure 2A).

**Table 1:** Survey-weighted baseline characteristics of individuals with stroke from NHANES.

Variables	Overall (*n* = 1,152)	Mortality (*n* = 539)	Survival (*n* = 613)	*p* value
Age, years	67 [58–77]	71 [61–79]	65 [55–75]	< 0.001
Gender, %				0.363
Female	57.7 (54.0–61.4)	55.9 (50.6–61.1)	59.3 (54.1–64.6)	
Male	42.3 (38.6–46.0)	44.1 (38.9–49.4)	40.7 (35.6–45.9)	
Race, %				0.106
Mexican American	4.5 (3.1–5.8)	4.7 (3.0–6.3)	4.3 (2.7–5.9)	
Other Hispanic	3.9 (2.4–5.4)	3.9 (1.7–6.2)	3.8 (2.2–5.5)	
Non-Hispanic White	68.6 (64.2–72.9)	72.4 (67.0–77.7)	65.3 (59.4–71.2)	
Non-Hispanic Black	16.3 (13.6–18.9)	14.1 (10.7–17.5)	18.1 (14.5–21.7)	
Other	6.6 (4.5–8.7)	4.7 (2.2–7.3)	8.2 (5.0–11.4)	
Education level, %				0.003
Below high school	12.9 (10.5–15.4)	17.8 (13.8–21.7)	8.8 (5.9–11.7)	
High school	48.2 (44.5–51.8)	46.7 (41.3–52.0)	49.4 (44.1–54.8)	
Above high school	38.8 (34.8–42.7)	35.4 (29.7–41.2)	41.6 (36.2–47.1)	
PIR	1.58 [1.00–2.83]	1.58 [1.04–2.71]	1.58 [0.97–2.95]	0.136
Smoking, %	55.8 (52.0–59.5)	55.7 (50.4–61.0)	55.9 (50.6–61.1)	0.956
Drinking, %	58.2 (54.6–61.9)	61.5 (56.4–66.7)	55.5 (50.3–60.6)	0.104
BMI	29.28 [25.40–34.34]	29.00 [25.27–33.97]	29.50 [25.57–34.57]	0.857
Hypertension, %	72.3 (68.8–75.7)	73.7 (68.9–78.5)	71.1 (66.2–76.0)	0.453
Diabetes mellitus, %	31.0 (27.6–34.4)	32.8 (27.8–37.8)	29.4 (24.9–34.0)	0.326
Hyperlipidemia, %	57.3 (53.6–61.1)	53.1 (47.8–58.4)	60.9 (55.7–66.1)	0.040
SBP, mmHg	134 [120–152]	137 [122–154]	130 [118–148]	0.022
DBP, mmHg	70 [60–78]	70 [59–78]	70 [60–78]	0.590
WBC, × 10^9^/L	7.20 [5.90–8.70]	7.10 [5.85–8.70]	7.30 [5.90–8.70]	0.817
Neutrophil, × 10^9^/L	4.30 [3.20–5.50]	4.30 [3.30–5.40]	4.30 [3.20–5.50]	0.528
Lymphocyte, × 10^9^/L	2.00 [1.50–2.50]	1.90 [1.50–2.40]	2.00 [1.60–2.60]	0.010
Monocyte, × 10^9^/L	0.60 [0.50–0.70]	0.60 [0.40–0.70]	0.60 [0.50–0.70]	0.666
Hemoglobin, g/dL	13.80 [12.70–14.82]	13.80 [12.70–15.00]	13.80 [12.70–14.70]	0.953
Platelet, × 10^9^/L	235 [198–282]	236 [200–284]	235 [196–281]	0.194
CRP, mg/L	3.00 [1.20–6.50]	3.20 [1.40–7.50]	2.70 [1.10–5.84]	0.037
Albumin, g/L	41.00 [39.00–43.00]	41.00 [39.00–44.00]	41.00 [39.00–43.00]	0.675
ALT, U/L	19.00 [15.00–25.00]	19.00 [15.00–24.00]	19.00 [14.00–26.00]	0.474
AST, U/L	22.00 [18.00–27.00]	22.00 [19.00–27.00]	22.00 [18.00–26.00]	0.670
eGFR, ml/min/1.73m^2^	70.61 [52.56–88.73]	67.44 [50.11–86.56]	73.66 [55.81–91.11]	< 0.001
HbA1c, %	5.80 [5.40–6.40]	5.80 [5.40–6.30]	5.80 [5.50–6.50]	0.606
TG, mmol/L	1.47 [0.92–2.25]	1.63 [1.08–2.39]	1.32 [0.85–2.13]	0.004
TC, mmol/L	4.86 [4.16–5.64]	4.99 [4.29–5.79]	4.76 [3.98–5.51]	0.010
LDL-C, mmol/L	2.76 [2.12–3.43]	2.84 [2.25–3.54]	2.61 [2.04–3.28]	0.005
HDL-C, mmol/L	1.24 [1.03–1.53]	1.22 [1.01–1.50]	1.27 [1.06–1.56]	0.174

PIR, poverty income ratio; BMI, body mass index; SBP, systolic blood pressure; DBP, diastolic blood pressure; WBC, white blood cell; CRP, C-reactive protein; ALT, alanine aminotransferase; AST, aspartate aminotransferase; eGFR, estimated glomerular filtration rate; HbA1c, glycated hemoglobin; TG, triglyceride; TC, total cholesterol; LDL-C, low-density lipoprotein cholesterol; HDL-C, high-density lipoprotein cholesterol.

**Table 2:** Comparisons of composite inflammatory indices between mortality and survival groups in NHANES and clinical cohorts.

NHANES cohort	Overall (*n* = 1,152)	Mortality (*n* = 539)	Survival (*n* = 613)	*p* value	Clinical cohort	Overall (*n* = 2,540)	Mortality (*n* = 289)	Survival (*n* = 2,251)	*p* value
SII	500.21 [354.97–724.11]	523.64 [381.69–770.73]	476.62 [334.44–688.07]	0.005	SII	699.50 [424.34–1255.32]	1213.89 [598.99–2245.82]	663.23 [415.46–1154.29]	< 0.001
NLR	2.16 [1.55–2.92]	2.26 [1.61–3.07]	2.05 [1.47–2.78]	0.008	NLR	3.81 [2.47–6.74]	6.94 [3.69–11.72]	3.61 [2.40–6.02]	< 0.001
PLR	120.00 [93.47–154.07]	125.78 [98.41–166.88]	114.48 [88.63–145.33]	< 0.001	PLR	135.97 [95.75–194.18]	172.89 [112.89–255.19]	132.52 [94.80–188.29]	< 0.001
LMR	3.50 [2.62–4.66]	3.33 [2.47–4.50]	3.66 [2.75–4.66]	0.008	LMR	2.94 [2.05–4.07]	2.24 [1.47–3.30]	3.03 [2.12–4.14]	< 0.001
NPR	0.018 [0.013–0.023]	0.018 [0.013–0.022]	0.018 [0.013–0.023]	0.809	NPR	0.029 [0.021–0.042]	0.040 [0.028–0.056]	0.028 [0.021–0.040]	< 0.001
SIRI	1.25 [0.79–1.83]	1.34 [0.82–1.90]	1.17 [0.75–1.80]	0.022	SIRI	1.75 [1.06–3.27]	3.12 [1.60–6.19]	1.66 [1.02–3.00]	< 0.001
PAR	5.73 [4.81–6.97]	5.72 [4.84–6.87]	5.75 [4.78–7.00]	0.360	PAR	4.56 [3.55–5.76]	4.79 [3.50–6.08]	4.54 [3.56–5.75]	0.271
NPAR	1.44 [1.28–1.63]	1.46 [1.29–1.67]	1.43 [1.26–1.61]	0.089	NPAR	1.78 [1.55–2.01]	2.01 [1.77–2.24]	1.75 [1.54–1.98]	< 0.001
CLR	1.44 [0.64–3.37]	1.66 [0.73–4.07]	1.27 [0.56–2.66]	0.008	CLR	1.06 [0.42–4.38]	2.91 [0.74–16.59]	0.94 [0.40–3.66]	< 0.001
CALLY	2.86 [1.21–6.44]	2.46 [0.94–5.47]	3.13 [1.49–7.43]	0.010	CALLY	3.81 [0.91–9.52]	1.44 [0.23–5.61]	4.32 [1.08–9.90]	< 0.001
PIV	294.76 [179.58–446.92]	312.09 [193.38–473.44]	274.81 [167.20–427.81]	0.018	PIV	319.94 [178.07–629.55]	566.86 [277.51–1306.23]	299.26 [173.23–577.24]	< 0.001
HALP	4.74 [3.46–6.28]	4.58 [3.28–5.98]	5.01 [3.60–6.60]	0.002	HALP	3.91 [2.64–5.73]	2.93 [1.90–4.43]	4.04 [2.75–5.85]	< 0.001

SII, systemic immune-inflammation index; NLR, neutrophil-to-lymphocyte ratio; PLR, platelet-to-lymphocyte ratio; LMR, lymphocyte-to-monocyte ratio; NPR, neutrophil-to-platelet ratio; SIRI, systemic immune-inflammation response index; PAR, platelet-to-albumin ratio; NPAR, neutrophil percentage-to-albumin ratio; CLR, C-reactive protein to lymphocyte ratio; CALLY, C-reactive protein-albumin-lymphocyte index; PIV, pan-immune-inflammation value; HALP, hemoglobin-albumin-lymphocyte-platelet score.

**Figure 3:**
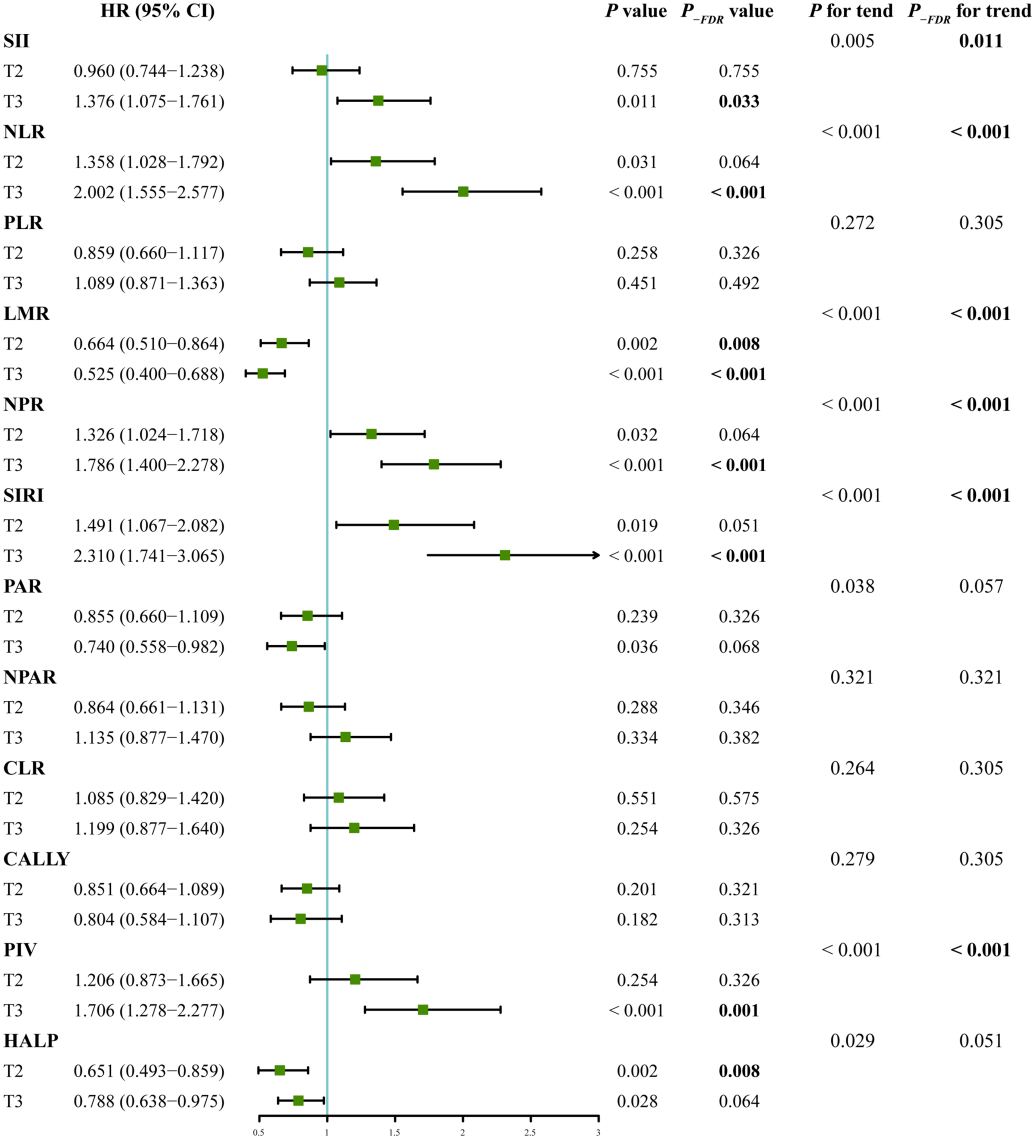
Forest plot exhibits the associations between tertiles of composite inflammatory indices and post-stroke mortality in NHANES cohort, using the lowest tertile as reference, and adjusting for age, sex, race, education level, smoking, drinking, body mass index, hypertension, diabetes mellitus, hyperlipidemia, estimated glomerular filtration rate, aspartate aminotransferase, triglyceride, total cholesterol, and low-density lipoprotein cholesterol.

**Figure 4:**
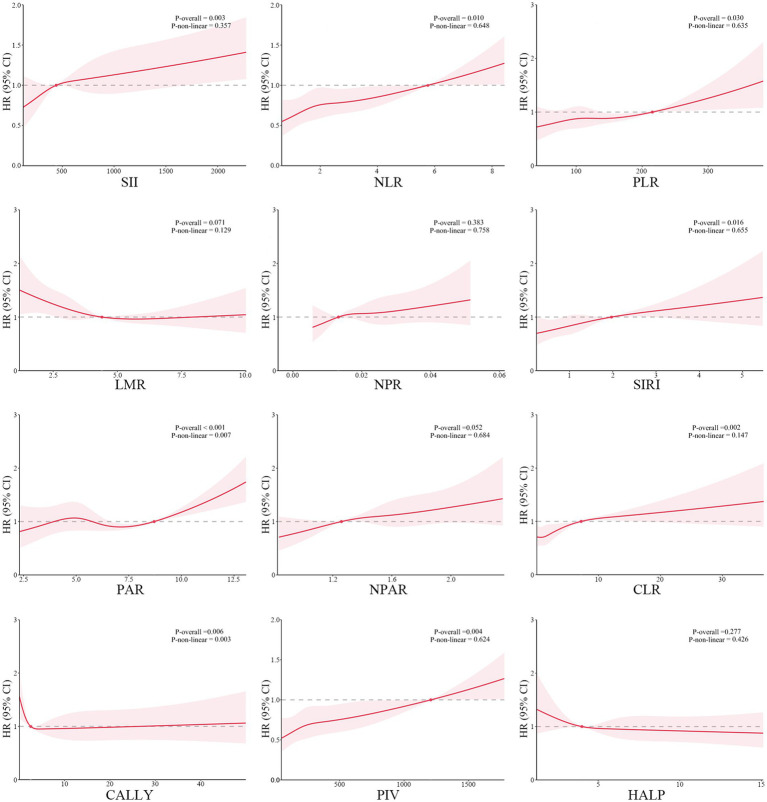
Restricted cubic spline for the association between composite inflammatory indices and post-stroke mortality in NHANES cohort after adjusting for age, sex, race, education level, smoking, drinking, body mass index, hypertension, diabetes mellitus, hyperlipidemia, estimated glomerular filtration rate, aspartate aminotransferase, triglyceride, total cholesterol, and low-density lipoprotein cholesterol.

**Figure 5:**
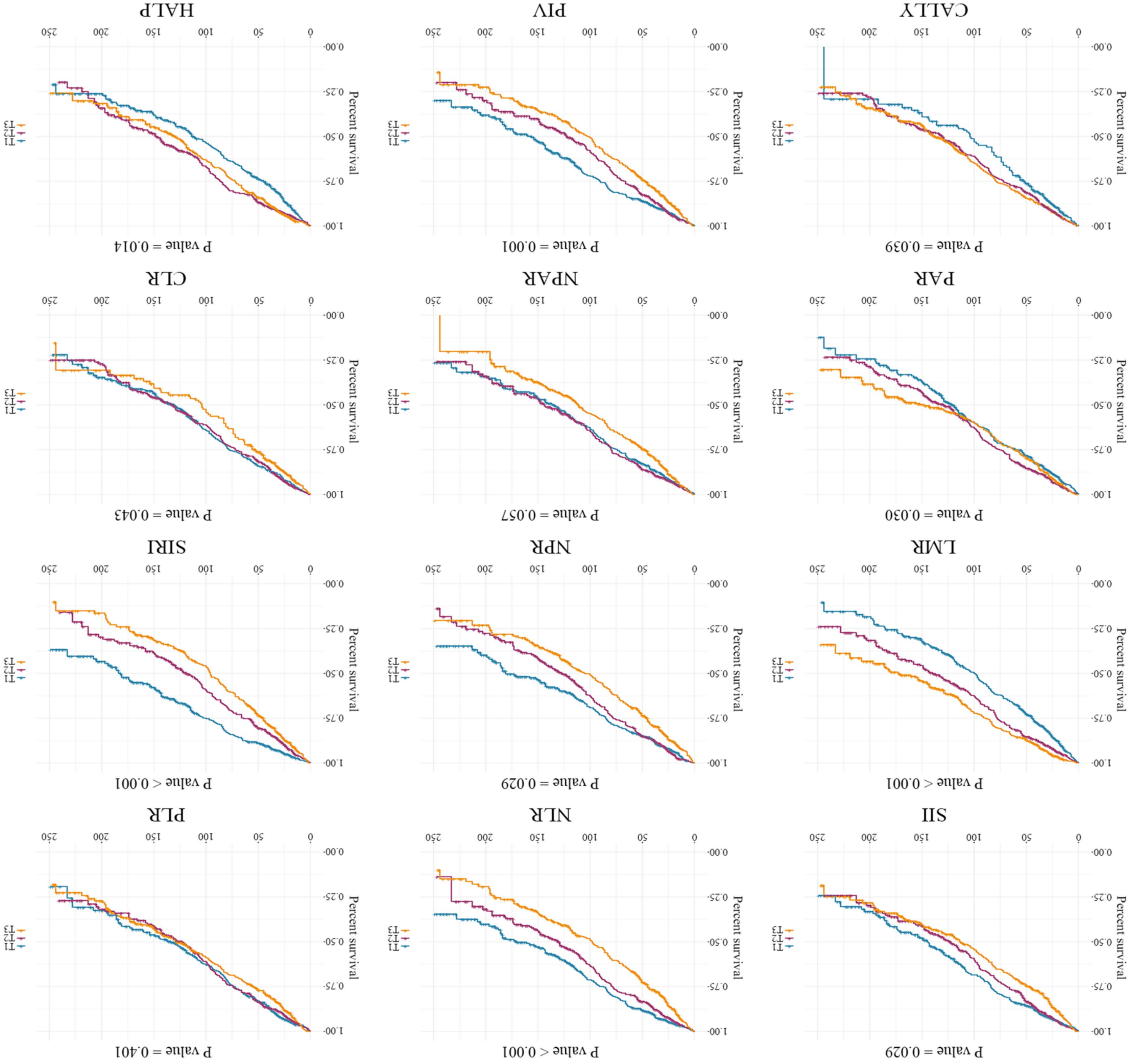
Kaplan–Meier curves indicate the relationship between tertiles of composite inflammatory indices and post-stroke mortality in NHANES cohort.

When analyzed as continuous variables, the weighted Cox proportional hazard modeling indicated that SII (per 100 unit, HR 1.020, 95% CI 1.008–1.033, *P_-FDR_* = 0.003), NLR (HR 1.101, 95% CI 1.043–1.163, *P_-FDR_* = 0.001), SIRI (HR 1.184, 95% CI 1.088–1.289, *P_-FDR_* = 0.001), and PIV (per 100 unit, HR 1.049, 95% CI 1.023–1.077, *P_-FDR_* = 0.001) were significantly associated with post-stroke mortality, after fully adjusting covariates in Model 3 (Table 3). When categorizing these four indices as ordinal variables, individuals in the highest tertiles had significantly increased risk of mortality (SII: HR 1.376, 95% CI 1.075–1.761, *P_-FDR_* = 0.033; NLR: HR 2.002, 95% CI 1.555–2.577, *P_-FDR_* < 0.001; SIRI: HR 2.310, 95% CI 1.741–3.065, *P_-FDR_* < 0.001; PIV: HR 1.706, 95% CI 1.278–2.277, *P_-FDR_* = 0.001), compared to those in the lowest tertiles (Figure 3). Furthermore, RCS curves demonstrated that the risk of mortality increased linearly with ascending SII (*P_-non-linear_* = 0.357), NLR (*P_-non-linear_* = 0.648), SIRI (*P_-non-linear_* = 0.655), and PIV (*P_-non-linear_* = 0.624) (Figure 4). These associations were also corroborated by Kaplan–Meier curves, which confirmed that elevated levels of these indices were associated with increased proportion of mortality (Figure 5).

**Table 3:** The associations between composite inflammatory indices and mortality.

NHANES cohort	Model 1^a^			Model 2^b^			Model 3^c^		
	HR (95% CI)	*p* value	*P*_-FDR_ value	HR (95% CI)	*p* value	*P*_-FDR_ value	HR (95% CI)	*p* value	*P*_-FDR_ value
SII^*^	1.026 (1.014–1.038)	<0.001	<0.001	1.024 (1.012–1.036)	<0.001	<0.001	1.020 (1.008–1.033)	0.001	0.003
NLR	1.115 (1.048–1.187)	<0.001	0.001	1.095 (1.031–1.162)	0.002	0.008	1.101 (1.043–1.163)	<0.001	0.001
PLR	1.002 (0.999–1.004)	0.072	0.096	1.001 (0.999–1.003)	0.068	0.081	1.001 (1.000–1.003)	0.028	0.056
LMR	0.898 (0.834–0.967)	0.004	0.009	0.956 (0.899–1.018)	0.160	0.174	0.947 (0.888–1.010)	0.098	0.118
NPR^^^	1.150 (1.028–1.286)	0.014	0.024	1.109 (0.995–1.238)	0.061	0.081	1.091 (0.974–1.221)	0.129	0.140
SIRI	1.246 (1.159–1.339)	<0.001	<0.001	1.179 (1.091–1.275)	<0.001	<0.001	1.184 (1.088–1.289)	<0.001	0.001
PAR	1.013 (0.955–1.075)	0.659	0.659	1.053 (0.999–1.110)	0.052	0.079	1.045 (0.992–1.101)	0.091	0.118
NPAR	1.835 (1.292–2.606)	<0.001	0.001	1.567 (1.141–2.152)	0.005	0.013	1.480 (1.044–2.097)	0.027	0.056
CLR	1.018 (1.002–1.033)	0.027	0.040	1.014 (1.001–1.029)	0.033	0.067	1.013 (0.999–1.026)	0.051	0.076
CALLY	0.989 (0.973–1.005)	0.184	0.200	0.994 (0.978–1.011)	0.527	0.527	0.998 (0.983–1.013)	0.822	0.822
PIV^*^	1.057 (1.032–1.084)	<0.001	<0.001	1.051 (1.027–1.077)	<0.001	<0.001	1.049 (1.023–1.077)	<0.001	0.001
HALP	0.961 (0.918–1.006)	0.091	0.109	0.966 (0.933–1.001)	0.052	0.079	0.964 (0.931–0.999)	0.045	0.076

Clinical cohort	Model 1^d^			Model 2^e^			Model 3^f^		
	HR (95% CI)	*p* value	*P*_-FDR_ value	HR (95% CI)	*p* value	*P*_-FDR_ value	HR (95% CI)	*p* value	*P*_-FDR_ value
SII^*^	1.010 (1.008–1.012)	<0.001	<0.001	1.014 (1.010–1.017)	<0.001	<0.001	1.005 (1.001–1.008)	0.019	0.042
NLR	1.045 (1.037–1.053)	<0.001	<0.001	1.04 (1.032–1.048)	<0.001	<0.001	1.023 (1.010–1.037)	<0.001	0.002
PLR	1.002 (1.001–1.002)	<0.001	<0.001	1.002 (1.001–1.002)	<0.001	<0.001	1.001 (1.000–1.001)	0.042	0.072
LMR	0.730 (0.667–0.800)	<0.001	<0.001	0.768 (0.701–0.840)	<0.001	<0.001	0.910 (0.840–0.986)	0.021	0.042
NPR^^^	1.093 (1.073–1.114)	<0.001	<0.001	1.086 (1.065–1.107)	<0.001	<0.001	1.034 (1.006–1.063)	0.017	0.042
SIRI	1.001 (1.001–1.002)	<0.001	<0.001	1.002 (1.001–1.002)	<0.001	<0.001	1.001 (1.001–1.002)	0.001	0.002
PAR	1.033 (0.980–1.090)	0.228	0.228	1.043 (0.991–1.098)	0.109	0.109	1.017 (0.956–1.081)	0.598	0.598
NPAR	2.532 (2.166–2.960)	<0.001	<0.001	2.13 (1.812–2.502)	<0.001	<0.001	1.272 (0.972–1.665)	0.079	0.118
CLR	1.002 (1.001–1.003)	<0.001	<0.001	1.003 (1.001–1.004)	<0.001	<0.001	1.001 (0.999–1.003)	0.585	0.598
CALLY	0.909 (0.885–0.934)	<0.001	<0.001	0.924 (0.900–0.949)	<0.001	<0.001	0.986 (0.961–1.011)	0.270	0.359
PIV^&^	1.010 (1.006–1.014)	<0.001	<0.001	1.013 (1.009–1.017)	<0.001	<0.001	1.009 (1.004–1.015)	0.001	0.002
HALP	0.870 (0.824–0.918)	<0.001	<0.001	0.906 (0.860–0.954)	<0.001	<0.001	0.978 (0.938–1.020)	0.308	0.369

* Per 100 units.

^ Per 0.01 unit.

& Per 1,000 units.

NHANES cohort: ^a^Model 1 adjusted no co-variable; ^b^Model 2 adjusted for age, sex, and race; ^c^Model 3 adjusted for age, sex, race, education level, smoking, drinking, BMI, hypertension, diabetes mellitus, hyperlipidemia, eGFR, AST, TG, TC, and LDL. Clinical cohort: ^d^Model 1 adjusted for no co-variable; ^e^Model 2 adjusted for age, sex, and time from onset to admission; ^f^Model 3 adjusted for age, sex, time from onset to admission, history of diabetes mellitus, coronary heart disease, atrial fibrillation and congestive heart failure, previous stroke, anticoagulant therapy at admission, TOAST classification, initial NIHSS score, sodium, chlorine, PT, fibrinogen, INR, AST, creatinine, cystatin C, and LDL-C. SII, systemic immune-inflammation index; NLR, neutrophil-to-lymphocyte ratio; PLR, platelet-to-lymphocyte ratio; LMR, lymphocyte-to-monocyte ratio; NPR, neutrophil-to-platelet ratio; SIRI, systemic immune-inflammation response index; PAR, platelet-to-albumin ratio; NPAR, neutrophil percentage-to-albumin ratio; CLR, C-reactive protein to lymphocyte ratio; CALLY, C-reactive protein-albumin-lymphocyte index; PIV, pan-immune-inflammation value; HALP, hemoglobin-albumin-lymphocyte-platelet score; HR, hazard ratio; CI, confidence interval; FDR, false discovery rate.

### 3.2 Clinical cohort

A total of 4,153 patients were initially assessed for eligibility at our institution, and 2,540 of them were finally included in this study (Figure 1B). By the follow-up, 289 (11.4%) patients had died after 90 days of onset. Compared to survival group, the mortality group was characterized by significantly older age, higher prevalence of female, higher initial NIHSS score, and greater burden of pre-existing medical conditions (Supplementary Table S1). Among the composite inflammatory indices, all except for PAR showed significant differences between the mortality and survival groups (all *p* < 0.05) (Table 2). The ROC curves showed that most indices possessed moderate discriminatory power for mortality, with NLR demonstrating the strongest discrimination ability (AUC = 0.703), followed by NPAR (AUC = 0.696), SIRI (AUC = 0.686), and SII (AUC = 0.674) (Figure 2).

**Figure 6:**
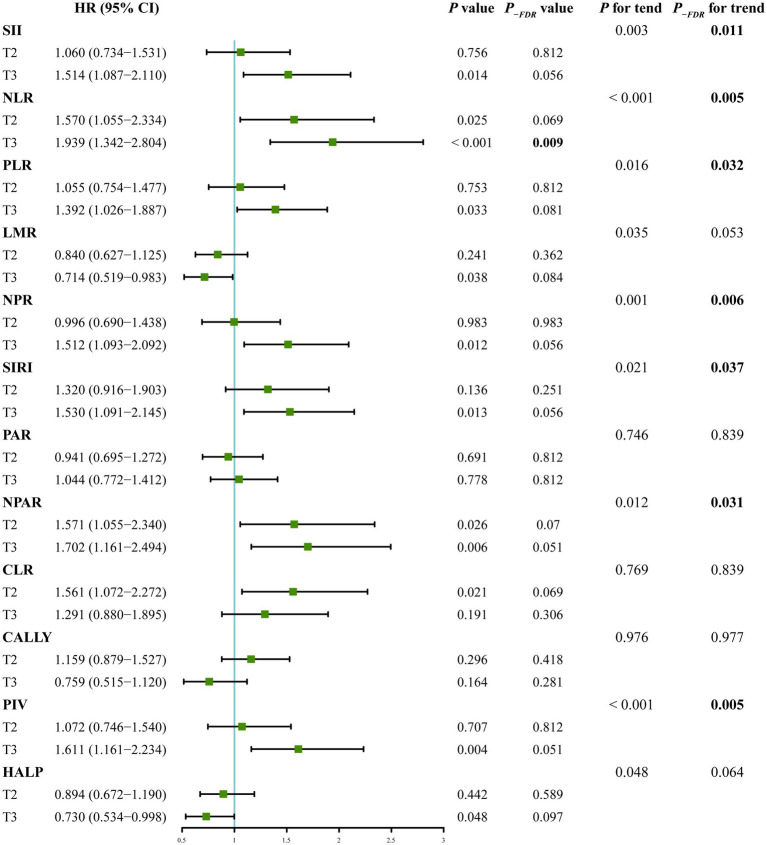
Forest plot exhibits the associations between tertiles of composite inflammatory indices and post-stroke mortality in clinical cohort, using the lowest tertile as reference, and adjusting for age, sex, time from onset to admission, history of diabetes mellitus, coronary heart disease, atrial fibrillation and congestive heart failure, previous stroke, anticoagulant therapy at admission, TOAST classification, initial NIHSS score, sodium, chlorine, prothrombin time, fibrinogen, international normalized ratio, aspartate aminotransferase, creatinine, cystatin C, and low-density lipoprotein cholesterol.

When treated as continuous variables, SII (per 100 unit, HR 1.005, 95% CI 1.001–1.008, *P_-FDR_* = 0.042), NLR (HR 1.023, 95% CI 1.010–1.037, *P_-FDR_* = 0.002), LMR (HR 0.910, 95% CI 0.840–0.986, *P_-FDR_* = 0.042), NPR (per 0.01 unit, HR 1.034, 95% CI 1.006–1.063, *P_-FDR_* = 0.042), SIRI (HR 1.001, 95% CI 1.001–1.002, *P_-FDR_* = 0.002), and PIV (per 1,000 unit, HR 1.009, 95% CI 1.004–1.015, *P_-FDR_* = 0.002) showed significant associations with post-stroke mortality, after fully adjustment of the covariates in Model 3 (Table 3). When categorized into tertiles, the association for NLR remained the most robust, with its highest tertile significantly associated with increased mortality risk (HR 1.939, 95% CI 1.342–2.804, *P_-FDR_* = 0.009, compared with the lowest tertile). While, the associations for the highest tertiles of SII, SIRI, LMR, NPR, and PIV were of marginal significance (*P*_-FDR_ = 0.056, 0.056, 0.084, 0.056 and 0.051, respectively) (Figure 6). The RCS curve also revealed a linear association between NLR level and mortality risk (*P_-non-linear_* = 0.132) (Supplementary Figure S1). This relationship was further confirmed by Kaplan–Meier curves, which demonstrated a significant correlation between elevated NLR tertiles and increased mortality (Supplementary Figure S2).

### 3.3 NLR is the most robust risk factor associated with mortality in both NHANES and clinical cohort

**Figure 7:**
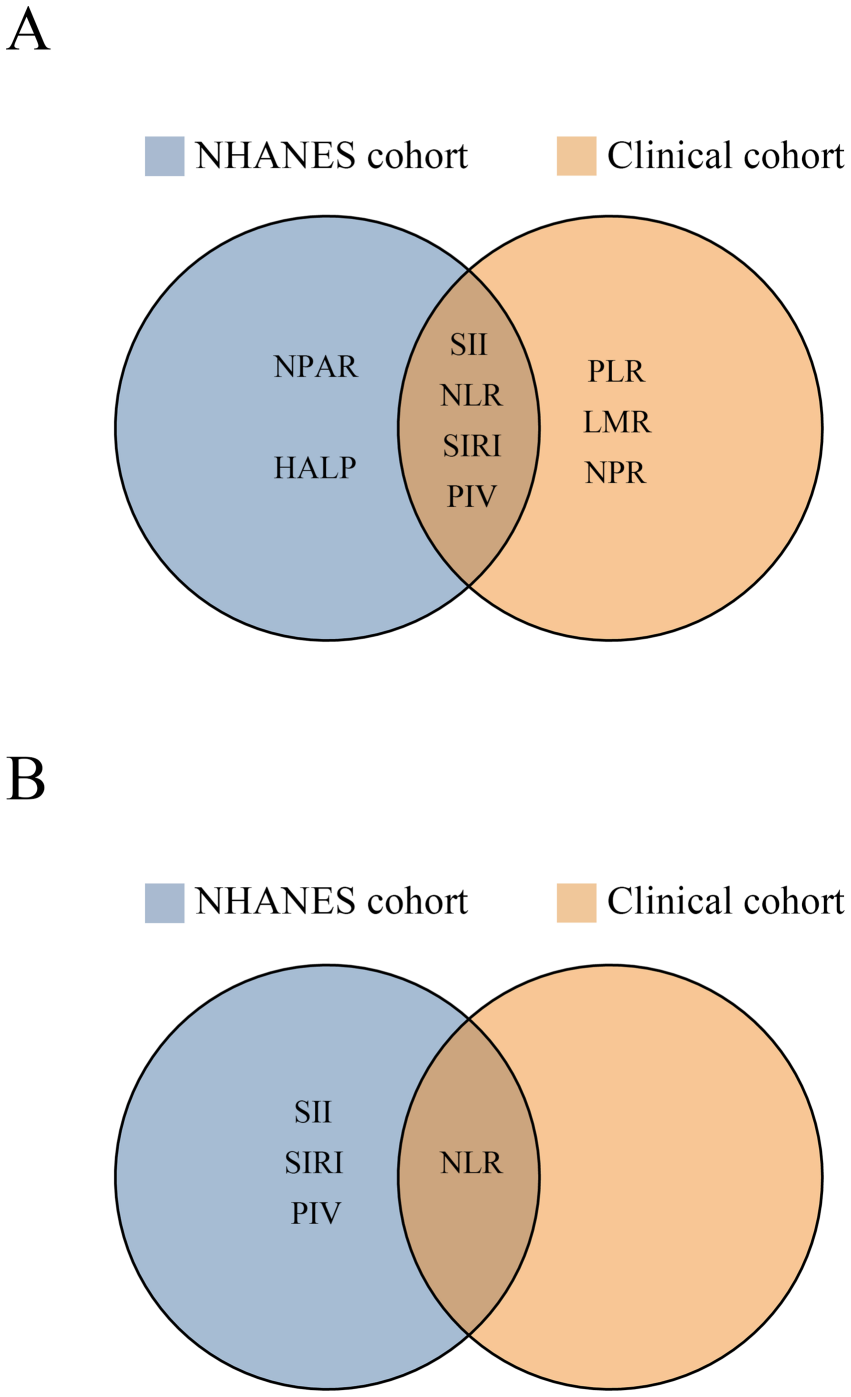
The composite inflammatory indices possessing significant association with post-stroke mortality in both NHANES and clinical cohorts. Before false discovery rate correction **(A)**; after false discovery rate correction **(B)**.

**Figure 8:**
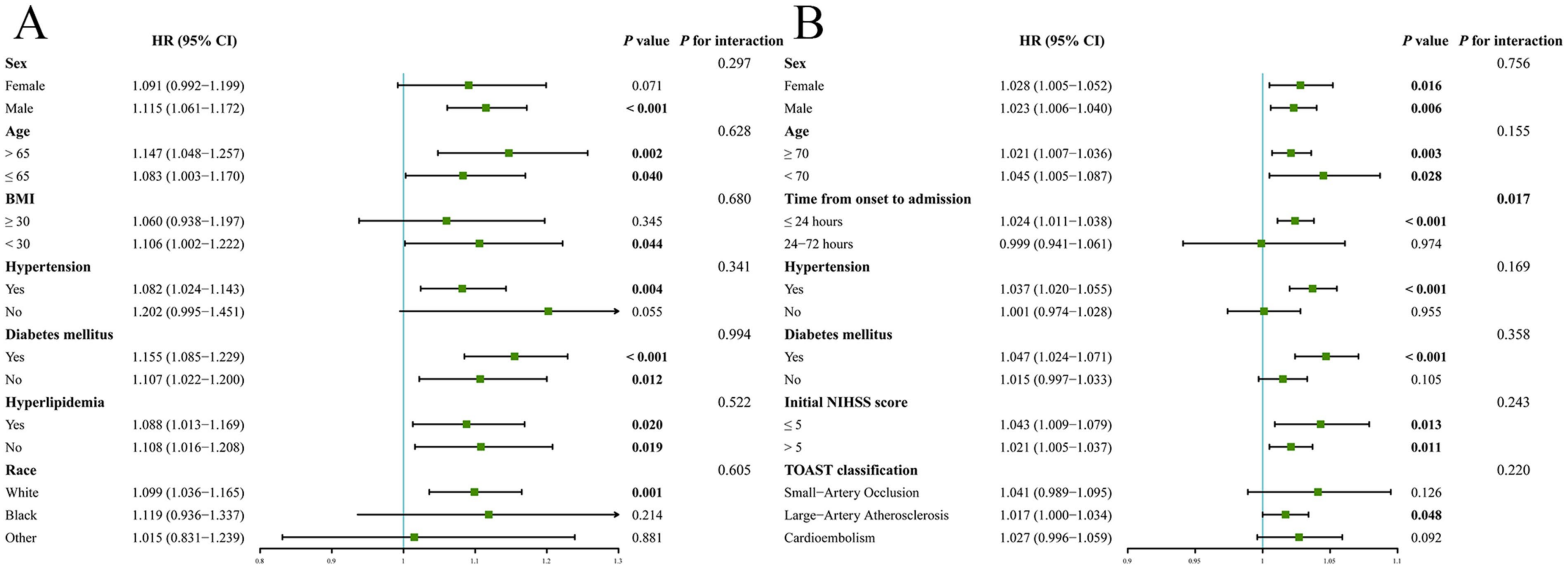
Subgroup analyses revealing the associations between neutrophil-to-lymphocyte ratio and post-stroke mortality under different conditions in NHANES **(A)** and clinical cohorts **(B)**.

Combining above results from the NHANES and clinical cohorts, we initially observed the SII, NLR, SIRI, and PIV were significantly associated with post-stroke mortality in both cohorts (Figure 7A). However, after FDR correction, NLR emerged as the most robust composite inflammatory index that retained significant association across both populations (Figure 7B). Therefore, we further explored the relationship between NLR and mortality in various scenarios through subgroup analyses. In the NHANES cohort, no significant interaction effects were observed between NLR and any of the stratification variables (all *P* for interaction > 0.05). Subgroup analyses revealed that the associations between NLR and mortality remained significant among male, individual with BMI < 30, and those of white race. Furthermore, this relationship was consistent across all age groups and irrespective of diabetes mellitus or hyperlipidemia status (all *p* < 0.05) (Figure 8A). In the clinical cohort, a significant interaction was noticed between NLR and time from onset to admission (*P* for interaction = 0.017) that NLR was linked to mortality in patients admitted within 24 h after AIS onset. Moreover, significant association was also identified among patients with history of hypertension and diabetes mellitus, and those with the large-artery atherosclerosis stroke subtype. In addition, this relationship was consistent across all categories of sex, age, and initial NIHSS score (all *p* < 0.05) (Figure 8B).

### 3.4 Incremental prognostic value of NLR

To evaluate the potential clinical utility of NLR, we assessed its incremental value when added to several baseline prognostic models in clinical cohort. The results were summarized in Table 4. When added to the Model 2, NLR significantly improved discrimination, with the C-statistic increasing from 0.708 to 0.767 (*p* < 0.001). The addition of NLR also yield substantial improvement in reclassification (category-free NRI = 61.8% [49.8–73.8%], *p* < 0.001) and integrated discrimination (IDI = 4.8% [3.2–6.4%], *p* < 0.001). Then, we evaluated the prognostic value of NLR in the Model 3, which demonstrated excellent discrimination (C-statistic = 0.909). Adding NLR led to a small but significant increase in the C-statistic to 0.912 (*p* = 0.049) and reclassification (category-free NRI = 34.1% [22.1–46.2%], *p* < 0.001), although the improvement in integrated discrimination was limited (IDI = 0.4% [−0.1–1.0%], *p* = 0.100). Finally, we used NIHSS score alone as the baseline. NLR also significantly improved the C-statistic (from 0.870 to 0.879, *p* = 0.003), reclassification (category-free NRI = 32.2% [20.2–44.1%], *p* < 0.001), and integrated discrimination (IDI = 0.7% [0.1–1.3%], *p* = 0.044).

**Table 4:** Reclassification and discrimination statistics for 90-day mortality by NLR among patients in clinical cohort.

Models	C-statistic		Category-free NRI		IDI	
	Estimate (95% CI)	*p* value	Estimate (95% CI), %	*p* value	Estimate (95% CI), %	*p* value
Model 2^a^	0.708 (0.677–0.739)	Reference		Reference		Reference
Model 2^a^ + NLR	0.767 (0.737–0.797)	<0.001	61.8 (49.8–73.8)	<0.001	4.8 (3.2–6.3)	<0.001
Model 3^b^	0.909 (0.891–0.926)	Reference		Reference		Reference
Model 3^b^ + NLR	0.912 (0.894–0.929)	0.049	34.1 (22.1–46.2)	< 0.001	0.4 (−0.1–1.0)	0.100
NIHSS score	0.870 (0.846–0.894)	Reference		Reference		Reference
NIHSS score + NLR	0.879 (0.857–0.901)	0.003	32.2 (20.2–44.1)	<0.001	0.7 (0.1–1.3)	0.044

a Model 2 adjusted for age, sex, and time from onset to admission.

b Model 3 adjusted for age, sex, time from onset to admission, history of diabetes mellitus, coronary heart disease, atrial fibrillation and congestive heart failure, previous stroke, anticoagulant therapy at admission, TOAST classification, initial NIHSS score, sodium, chlorine, PT, fibrinogen, INR, AST, creatinine, cystatin C, and LDL-C.

NIHSS, National Institute of Health Stroke Scale; NLR, neutrophil-to-lymphocyte ratio; NRI, net-reclassification index; IDI, integrated discrimination improvement.

## 4 Discussion

In this study, we comprehensively evaluated 12 common composite indices across community and clinical cohorts to identify generalizable inflammatory markers for stroke mortality. Initial analysis of the NHANES cohort confirmed SII, NLR, SIRI, and PIV linked to mortality in stroke participants. However, in the clinical cohort, NLR maintained consistent association with mortality after FDR correction, whether treated as a continuous or categorical variable. Therefore, our study firstly proposes NLR as a generalizable inflammatory marker for post-stroke mortality in both population-based and clinical conditions.

Substantial literature implicates that the composite inflammatory indices, derived from peripheral blood parameters, are closely associated with the occurrence, progression, complications, and prognosis of stroke (Ye et al., 2025; Lee et al., 2024; Lv et al., 2023; Qian et al., 2024). Among these, the NLR has also been extensively investigated. Wu et al. reported that NLR levels measured both at 24 h and 12 days after intravenous thrombolysis were independently associated with 3-month mortality in patients with AIS (Wu and Chen, 2023). Recently, Xu et al. reported that NLR is correlated with both stroke morbidity and mortality (Xu et al., 2025). Our findings corroborate and extend prior findings by demonstrating that an elevated NLR level reflects increased risk of mortality not only in community-based stroke participants, but also in clinical AIS patients, thereby bridging epidemiological observations and clinical patient outcomes.

A critical consideration in interpreting these consistent findings is fundamental biological heterogeneity between the two cohorts, which represent distinct phases of stroke with different mechanisms linking to mortality. In the NHANES cohort, comprised of community-based population, mortality risk is assessed in a chronic phase. Here, an elevated NLR level likely signifies a chronic and low-grade systemically inflammatory status, potentially driven by atherosclerosis, or age-related immunosenescence (Simats and Liesz, 2022; Chen et al., 2024b). This status progressively depletes physiological reserve and worsens vascular health, creating a substrate for higher long-term mortality after a stroke event (Sykes et al., 2021). In contrast, within the AIS cohort capturing acute-phase mortality, the elevated NLR primarily represents an acute and stress-induced inflammatory burst, which may promote endothelial dysfunction, disrupt blood–brain barrier (BBB), facilitate platelet activation, and aggravate microvascular occlusion (Xu et al., 2025; Esenwa and Elkind, 2016; Iadecola and Anrather, 2011). Specifically, in the early phase of AIS, neutrophils infiltrate the ischemic brain tissue to exacerbate cellular injury and extracellular matrix damage through the release of reactive oxygen species, proteases, and proinflammatory factors (Li et al., 2022; Zhang et al., 2022). In contrast, lymphocytes function as primary neuroprotective immunoregulators. Specific lymphocyte subpopulations help preserve immune homeostasis and mitigate cerebral damage by limiting the production of proinflammatory mediators, regulating the activation of autoreactive immune cells, and fostering neural repair processes (Lattanzi et al., 2021). This neuroprotective capacity, however, is compromised by a stroke-induced systemic stress response, characterized by elevated catecholamines and cortisol, which trigger apoptosis and functional suppression of peripheral lymphocytes (Lattanzi et al., 2021). Therefore, the short-term risk of mortality appears to be propelled by a dual pathology of destructive inflammation (high neutrophil level) and immune suppression (low lymphocyte level). Despite these profound differences in the underlying pathophysiological mechanisms, chronic low-grade inflammation versus inflammatory/immunosuppressive stress, NLR emerged as a consistent prognostic biomarker in both cohorts, suggesting that NLR may capture a common pathway of inflammatory dysregulation that is deleterious across the stroke continuum, from acute injury to long-term survival. Our multicohort design, therefore, not only validated NLR in diverse settings but highlighted its unique utility in transcending specific disease stages to signal an elevated risk of mortality.

Further detailed analyses of NLR demonstrated a significant interaction between NLR and the time from onset to admission in patients with AIS. Stratified analysis revealed that the association was specific to patients admitted within 24 h after AIS onset. The specificity of this association to the first 24 h after onset aligns with the early-phase dynamics of the innate immune response in AIS. This hyper-acute window is characterized by a rapid and pronounced neutrophilic surge, driven by the release of damage-associated molecular patterns (DAMPs) from ischemic tissue (Wang et al., 2024; Amini et al., 2023; Thomas et al., 2021). Neutrophils are the earliest leukocyte adhering to the activated endothelium, releasing reactive oxygen species, and initiating early infiltrated processes that directly exacerbate BBB disruption and secondary brain injury (Li et al., 2022; Zhang et al., 2022). Concurrently, a systemic stress response, mediated by catecholamines and cortisol, induces rapid lymphocyte apoptosis and functional immunosuppression (Qian et al., 2024). Thus, an elevated NLR measured within this narrow time frame may capture the peak imbalance between the pro-inflammatory neutrophilic drive and the concomitant loss of immunoregulatory lymphocyte function. At the later time window of AIS, the immune response transitions into a more complex and regulated phase involving lymphocyte subset reconstitution and the resolution of inflammation (Lukasik et al., 2023). Therefore, a single NLR measurement at later time point may less accurately reflect the initial inflammatory insult that may account for the attenuated association observed in patients admitted in later time window. The characteristics of patients in our clinical cohort also provided a plausible explanation of this finding. High proportion of patients with minor stroke (like small artery occlusion subtype) were enrolled into this study that involves a comparatively modest and transient systemic inflammatory response. As time elapsed after AIS onset, the inflammatory response stabilized through intrinsic regulation, further contributing to the attenuation of correlation between NLR and mortality in patients admitted within later time window (Pacinella et al., 2025).

Several immune–inflammation indicators related with stroke prognosis have been proposed previously (Qian et al., 2024; Altuntaş et al., 2025). Consistently, we also identified multiple composite inflammatory indices, including SII, NLR, SIRI, and PIV, that were correlated with risk of mortality after stroke in both the NHANES and clinical cohorts prior to FDR correction. Notably, in the continuous variable models, SII, SIRI, and PIV demonstrated significant associations with mortality even after rigorous FDR correction, underscoring their potential relevance in assessing inflammatory burden. The aim of our study, however, was not merely to re-affirm these associations or dispute the established relevance of these markers as potential predictors. Rather, our objective was to distinguish the most robust inflammatory factor that performs reliably for assessing stroke mortality risk across diverse real-world settings, from community populations to clinical patients, through rigorous statistical methods. The selection of NLR as a consistent composite inflammatory index can be attributed to two factors. From the biological perspective, NLR corporates two of the most fundamental cellular parameters in the inflammatory response: neutrophil (representing innate immune and pro-inflammatory forces) and lymphocyte (reflecting adaptive and regulatory immune functions). Methodologically, in the complex and dynamic inflammatory milieu following stroke, indices incorporating an excessive number of parameters may be susceptible to large fluctuations and reduced stability. The relative simplicity and biological consonance of NLR likely underpin its robustness as a consistent related inflammatory index of mortality risk across both community and clinical conditions.

Our multicohort design confirmed NLR as a highly consistent biomarker of mortality after stroke. However, its clinical utility as an independent predictive tool was constrained by its moderate discriminative capacity, a finding that necessitated a nuanced interpretation of “robustness.” To evaluate the clinical utility of NLR, we assessed its incremental prognostic value when combined with established models. These analyses addressed the distinction between statistical association and clinical utility. Our findings indicated that NLR provided incremental prognostic information, especially in terms of risk reclassification. These findings suggested that NLR could be employed as a supplementary biomarker within a multifactorial risk stratification framework, aiding in identification of individuals at high or low risk relative to their clinical presentation.

Although a significant relationship between NLR and risk of mortality after stroke was observed in this study, its utility for guiding real-time clinical decisions remains limited at present, as only a statistical correlation has been identified. Nevertheless, our findings substantiate that, among numerous composite inflammatory indices, NLR emerges as the most robust candidate for assessing mortality risk after stroke, meriting further investigation into its clinical application.

This study has several strengths. First, the concurrent use of the NHANES database and a relatively large clinical cohort enhances the external validity of our findings, ensuring the results are not limited to a single population but are applicable to both community individuals and clinical patients. Furthermore, the rigorous statistical methods employed in the analysis of composite inflammatory indices, such as FDR correction for multiple comparisons, strengthens the robustness and reliability of the findings. Certainly, this study has several limitations. First, the cross-sectional nature of NHANES data and the retrospective design of the clinical cohort can only indicate statistical correlation rather than causality, and interpretation should be combined with practice. Second, the data of comorbidities in NHANES database and medical history in clinical cohort largely relied on self-report, which is susceptible to recall bias. Third, although we adjusted for a broad range of covariates, residual confounding due to unmeasured or unrecorded variables remains possible, such as medical use (e.g., statins, or anti-inflammatory agents), detailed nutritional status, post-stroke complications, and rehabilitation interventions, all of which could potentially influence both systemic inflammation and mortality risk. The absence of these data precluded fully disentangling the independent effect of the composite inflammatory indices from these influences. Therefore, while models provided robust evidence of association, the results should be interpreted with caution. Fourth, the specific stroke subtype could not be identified in NHANES database. Given the high prevalence of ischemic stroke and its closer links to chronic inflammation, most stroke participants in our study likely had ischemic stroke (Mao et al., 2024). However, the inclusion of unclassified stroke events, encompassing both ischemic and hemorrhagic strokes, in a single analytical framework introduces pathophysiological heterogeneity. Furthermore, even within ischemic stroke, different etiological subtype (for example, by TOAST classification) may exhibit variations in their systemic immune responses. The complete absence of these data in the NHANES cohort thus compounds the heterogeneity within our analytical sample. This heterogeneity may result in non-differential misclassification, which likely attenuated the observed association between composited inflammatory indices and mortality, causing conservative estimates of their true prognostic strength within a more homogeneous population (Brenner et al., 1993). While the findings in the clinical cohort supported the relevance of NLR in the ischemic subtype, the results in the NHANES cohort only suggested that an elevated NLR may capture a heightened inflammatory state that portends poorer survival in different stroke mechanisms, rather than establish it as a universal biomarker equally applicable across all stroke subtypes. Future studies with rigorous stroke subtype data are essential to validate the application of NLR in precision prognosis. Fifth, regarding the clinical cohort, the exclusion of patients who underwent MT or DC limited the generalizability of our findings, although this decision was made primarily to control for major confounding. Both MT and DC are high-intensity interventions that drastically alter the natural history of stroke. Critically, these procedures provoke profound inflammatory and stress response, which would substantially confound the interpretation of baseline composite inflammatory indices. By excluding these patients, we prioritized to delineate the associations between baseline composite inflammatory indices and mortality in patients managed with medical or standard supportive care. However, this protocol indeed introduced selection bias and reduced the representativeness of our sample with respect to the broader spectrum of real-world AIS patients. Therefore, our findings derived from clinical cohort primarily applied to patients with mild-to-moderate severity of stroke. Finally, the single center data of clinical cohort may limit the generalization of our clinical findings. A prospective multicenter study is needed to validate our findings.

## 5 Conclusion

In conclusion, our study provides a comprehensive evaluation of the association between 12 common composite inflammatory indices and post-stroke mortality risk, highlighting the NLR as a generalizable biomarker of mortality assessment across both community and clinical settings. Moreover, in the clinical practice, NLR demonstrates an interaction with time from onset to admission, revealing the correlation with mortality is pronounced in patients during early stage of AIS, and underscoring its time-sensitive prognostic value.

## Data Availability

The datasets presented in this study can be found in online repositories. The names of the repository/repositories and accession number(s) can be found at: the data analyzed in the current study are freely accessible on the NHANES website (https://www.cdc.gov/nchs/nhanes/index.htm), and are available from the corresponding author upon reasonable request.

## Footnotes

1
https://www.cdc.gov/nchs/nhanes/irba98.htm

2
https://www.cdc.gov/nchs/data_access/restrictions.htm

## Supplementary material

Supplementary Figure S1 Restricted cubic spline for the association between composite inflammatory indices and post-stroke mortality in clinical cohort after adjusting for age, sex, time from onset to admission, history of diabetes mellitus, coronary heart disease, atrial fibrillation and congestive heart failure, previous stroke, anticoagulant therapy at admission, TOAST classification, initial NIHSS score, sodium, chlorine, PT, fibrinogen, INR, AST, creatinine, cystatin C, and LDL-C.

Supplementary Figure S2 Kaplan–Meier curves indicate the relationship between tertiles of composite inflammatory indices and post-stroke mortality in clinical cohort.
