# Supplementary figures and images for: A comprehensive evaluation of the associations between 12 composite inflammatory indices and all-cause mortality after stroke: a multicohort study
Source: Front Aging Neurosci. 2026 Jan 9;17:1754095. doi: 10.3389/fnagi.2025.1754095 (PMC12827790; doi:10.3389/fnagi.2025.1754095)

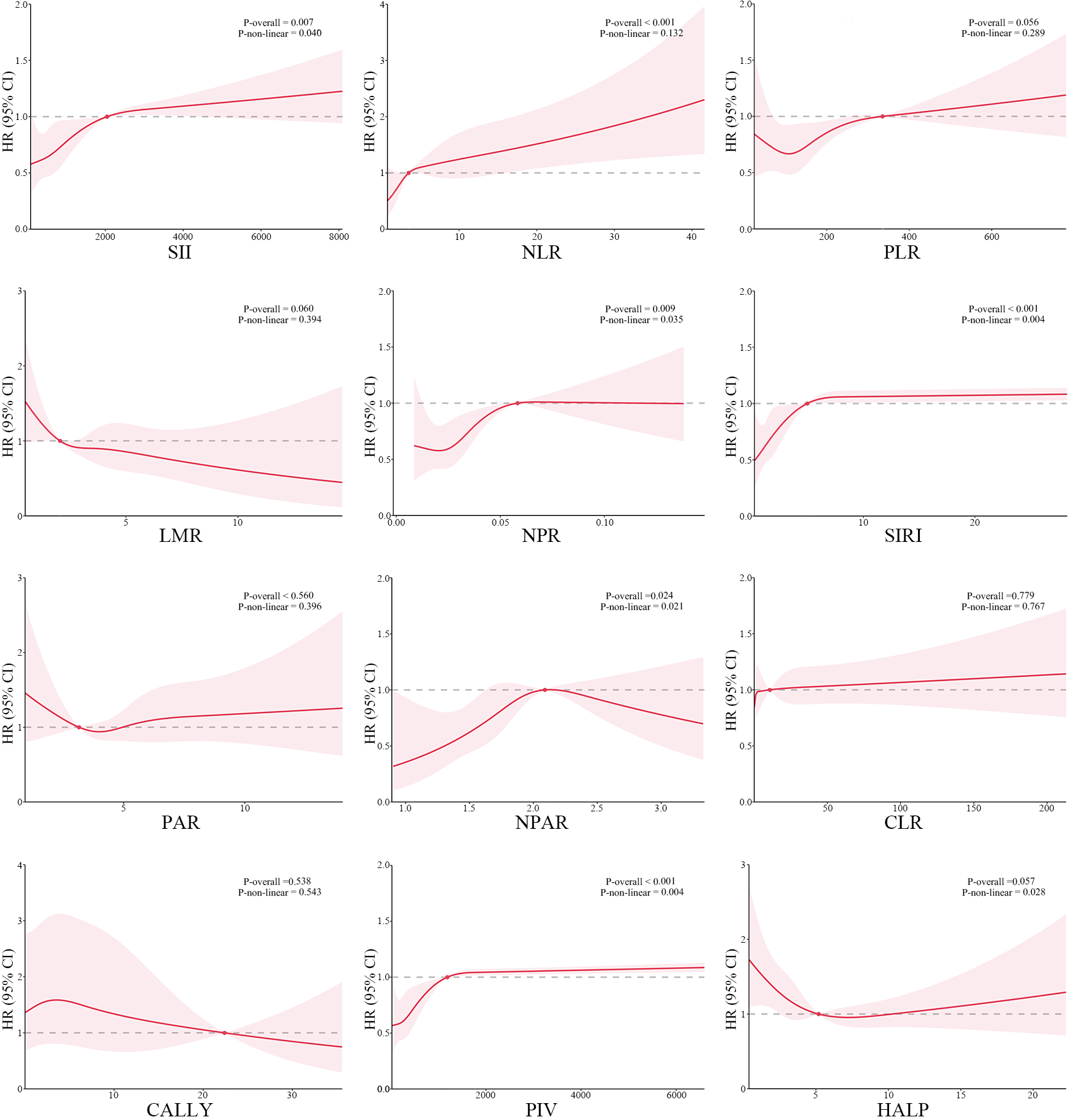

Supplement: Supplementary Figure S1 — Restricted cubic spline for the association between composite inflammatory indices and post-stroke mortality in clinical cohort after adjusting for age, sex, time from onset to admission, history of diabetes mellitus, coronary heart disease, atrial fibrillation and congestive heart failure, previous stroke, anticoagulant therapy at admission, TOAST classification, initial NIHSS score, sodium, chlorine, PT, fibrinogen, INR, AST, creatinine, cystatin C, and LDL-C. [file Image_1.tif]

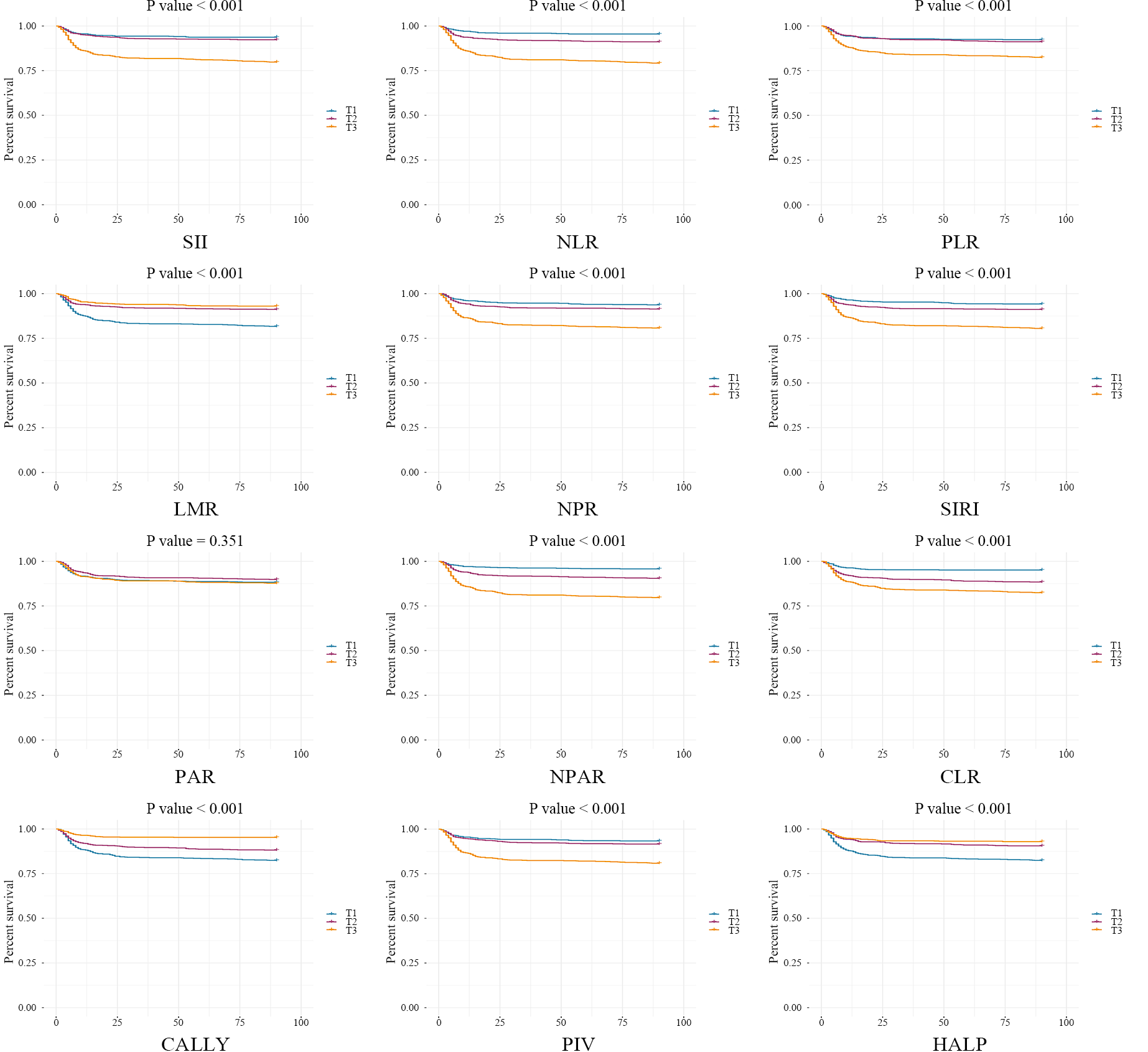

Supplement: Supplementary Figure S2 — Kaplan–Meier curves indicate the relationship between tertiles of composite inflammatory indices and post-stroke mortality in clinical cohort. [file Image_2.tif]
