# Supplementary material for: A comprehensive evaluation of the associations between 12 composite inflammatory indices and all-cause mortality after stroke: a multicohort study
Source: Front Aging Neurosci. 2026 Jan 9;17:1754095. doi: 10.3389/fnagi.2025.1754095 (PMC12827790; doi:10.3389/fnagi.2025.1754095)
Supplement: Supplementary file 3 [file Table_1.docx]

Supplementary Table S1. Baseline characteristics of patients with or without mortality at 90-day follow-up in clinical cohort.

| Variables | Overall (n=2540) | Mortality (n=289) | Survival (n=2251) | P value |
| --- | --- | --- | --- | --- |
| Age, year | 72 [65-79] | 78 [71-84] | 71 [63-78] | < 0.001 |
| Female, n (%) | 1060 (41.7) | 155 (53.6) | 905 (40.2) | < 0.001 |
| Time from onset to admission, n (%) |  |  |  | < 0.001 |
| ≤ 24 hours | 1916 (75.4) | 257 (88.9) | 1659 (73.7) |  |
| 24-48 hours | 413 (16.3) | 24 (8.3) | 389 (17.3) |  |
| 48-72 hours | 211 (8.3) | 8 (2.8) | 203 (9.0) |  |
| Intravenous thrombolysis, n (%) | 421 (16.6) | 59 (20.4) | 362 (16.1) | 0.062 |
| Medical history, n (%) |  |  |  |  |
| Hypertension | 1570 (61.8) | 179 (61.9) | 1391 (61.8) | 0.962 |
| Diabetes mellitus | 729 (28.7) | 105 (36.3) | 624 (27.7) | 0.002 |
| Coronary heart disease | 223 (8.8) | 38 (13.1) | 185 (8.2) | 0.005 |
| Atrial fibrillation | 494 (19.4) | 98 (33.9) | 396 (17.6) | < 0.001 |
| Rheumatic heart disease | 79 (3.1) | 14 (4.8) | 65 (2.9) | 0.071 |
| Congestive heart failure | 67 (2.6) | 17 (5.9) | 50 (2.2) | < 0.001 |
| Previous stroke | 144 (5.7) | 28 (9.7) | 116 (5.2) | 0.002 |
| Anticoagulant therapy at admission, n (%) | 63 (2.5) | 15 (5.2) | 48 (2.1) | 0.002 |
| Antiplatelet therapy at admission, n (%) | 64 (2.5) | 7 (2.4) | 57 (2.5) | 0.911 |
| Oral statin at admission, n (%) | 63 (2.5) | 10 (3.5) | 53 (2.4) | 0.255 |
| TOAST classification, n (%) |  |  |  | < 0.001 |
| Small-Artery Occlusion | 1133 (44.6) | 23 (8.0) | 1110 (49.3) |  |
| Large-Artery Atherosclerosis | 777 (30.6) | 139 (48.1) | 638 (28.3) |  |
| Cardioembolism | 535 (21.1) | 114 (39.4) | 421 (18.7) |  |
| Other Determined Etiology | 48 (1.9) | 4 (1.4) | 44 (2.0) |  |
| Undetermined Etiology | 47 (1.9) | 9 (3.1) | 38 (1.7) |  |
| Smoke, n (%) | 519 (20.4) | 71 (24.6) | 448 (19.9) | 0.064 |
| Alcoholism, n (%) | 347 (13.7) | 49 (17.0) | 298 (13.2) | 0.083 |
| SBP, mmHg | 146 [128-163] | 147 [128-165] | 146 [128-163] | 0.720 |
| DBP, mmHg | 84 [74-94] | 83 [73-93] | 84 [74-94] | 0.482 |
| Initial NIHSS score | 5 [2-10] | 16 [11-21] | 4 [2-8] | < 0.001 |
| Glucose at admissio, mmol/L | 7.32 [6.04-9.88] | 7.85 [6.40-10.10] | 7.30 [6.00-9.80] | 0.016 |
| Sodium, mmol/L | 139.90 [137.90-141.80] | 139.00 [137.02-141.07] | 140.00 [138.00-141.90] | < 0.001 |
| Potassium, mmol/L | 3.76 [3.49-4.02] | 3.78 [3.40-4.10] | 3.76 [3.50-4.02] | 0.636 |
| Chlorine, mmol/L | 104.50 [102.00-106.80] | 103.65 [100.60-106.00] | 104.60 [102.20-106.80] | < 0.001 |
| CRP, mg/L | 1.46 [0.50-5.19] | 3.05 [0.68-14.26] | 1.27 [0.50-4.73] | < 0.001 |
| WBC, × 10^9^/L | 7.60 [6.02-9.60] | 9.20 [6.90-11.80] | 7.40 [6.00-9.30] | < 0.001 |
| Neutrophil, × 10^9^/L | 5.30 [3.97-7.39] | 7.31 [5.00-9.96] | 5.15 [3.91-7.03] | < 0.001 |
| Lymphocyte, × 10^9^/L | 1.34 [0.95-1.82] | 1.11 [0.73-1.49] | 1.38 [0.99-1.85] | < 0.001 |
| Monocyte, × 10^9^/L | 0.47 [0.36-0.61] | 0.50 [0.35-0.65] | 0.46 [0.36-0.61] | 0.054 |
| Hemoglobin, g/dL | 13.30 [12.12-14.40] | 12.80 [11.80-13.80] | 13.30 [12.20-14.40] | < 0.001 |
| Platelet, × 10^9^/L | 186 [143-231] | 185 [139-236] | 186 [143-230] | 0.953 |
| PT, s | 12.10 [11.00-13.00] | 12.30 [11.32-13.37] | 12.10 [11.00-13.00] | < 0.001 |
| TT, s | 17.00 [16.10-18.00] | 16.90 [15.90-17.90] | 17.00 [16.20-18.00] | 0.101 |
| APTT, s | 30.70 [27.50-35.40] | 29.70 [26.80-34.20] | 30.80 [27.60-35.60] | 0.007 |
| Fibrinogen, g/L | 3.11 [2.62-3.68] | 3.28 [2.62-4.04] | 3.10 [2.63-3.65] | 0.020 |
| INR | 0.97 [0.92-1.03] | 1.01 [0.96-1.08] | 0.96 [0.91-1.02] | < 0.001 |
| Albumin, g/L | 40.50 [37.80-43.10] | 39.65 [36.80-41.97] | 40.6 [37.80-43.20] | < 0.001 |
| ALT, U/L | 18 [13-26] | 18 [13-26] | 18 [13-26] | 0.864 |
| AST, U/L | 24 [19-30] | 27 [21-34] | 23 [19-29] | < 0.001 |
| Creatinine, ummol/L | 71.30 [58.32-88.20] | 74.30 [58.70-94.75] | 71.00 [58.20-88.00] | 0.015 |
| Cystatin C, mg/L | 1.08 [0.91-1.31] | 1.15 [0.94-1.48] | 1.07 [0.91-1.30] | < 0.001 |
| Uric acid, ummol/L | 336.00 [272.00-407.00] | 331.50 [265.50-424.75] | 337.00 [273.00-406.00] | 0.928 |
| TG, mmol/L | 1.17 [0.78-1.76] | 1.31 [1.00-1.78] | 1.14 [0.75-1.76] | < 0.001 |
| TC, mmol/L | 4.77 [3.93-5.57] | 4.89 [3.90-5.56] | 4.75 [3.39-5.57] | 0.419 |
| LDL, mmol/L | 2.78 [2.22-3.39] | 2.97 [2.33-3.49] | 2.76 [2.21-3.36] | 0.011 |
| HDL, mmol/L | 1.30 [1.10-1.54] | 1.34 [1.14-1.61] | 1.29 [1.10-1.54] | 0.029 |
| Apo A1, g/L | 1.34 [1.18-1.51] | 1.33 [1.16-1.52] | 1.34 [1.18-1.51] | 0.770 |
| Apo B, g/L | 0.93 [0.76-1.13] | 0.92 [0.74-1.13] | 0.93 [0.76-1.13] | 0.492 |
| HbA1c, % | 6.20 [5.80-7.10] | 6.20 [5.80-7.00] | 6.20 [5.80-7.10] | 0.498 |

TOAST: Trail of ORG 10172 in Acute Stroke Treatment; SBP: systolic blood pressure; DBP: diastolic blood pressure; NIHSS: National Institute of Health Stroke Scale; CRP: C-reactive protein; WBC: white blood cell; PT: Prothrombin time, TT: Thrombin time, APTT: Activated partial thromboplastin time, INR: International normalized ratio, TG: Triglyceride, TC: Total cholesterol,HDL-C: High-density lipoprotein cholesterol, LDL-C: Low-density lipoprotein cholesterol; HbA1c: glycated hemoglobin; Apo: apolipoprotein.
